# Adaptation of an Evidence-Based Colorectal Cancer Screening Program Using the Consolidated Framework for Implementation Research

**DOI:** 10.5888/pcd12.150300

**Published:** 2015-12-03

**Authors:** Allison M. Cole, Andrea Esplin, Laura-Mae Baldwin

**Affiliations:** Author Affiliations: Andrea Esplin, Family Medicine Residency of Idaho, Boise, Idaho; Laura-Mae Baldwin, University of Washington, Seattle, Washington.

## Abstract

**Introduction:**

Federally Qualified Health Centers (FQHCs) provide primary care to low-income and uninsured patients in the United States. FQHCs are required to report annual measurements and provide evidence of improvement for quality measures; effective methods to improve quality in FQHCs are needed. Systems of Support (SOS) is a proactive, mail-based, colorectal cancer screening program that was developed and tested in an integrated health care system. The objective of this study was to adapt SOS for use in an FQHC system, guided by the Consolidated Framework for Implementation Research (CFIR).

**Methods:**

We conducted qualitative semi-structured interviews in 2014 with organizational leadership, medical staff, and nursing staff to identify facilitators of and barriers to implementation of SOS in an FQHC system. The interview guide was based on the CFIR framework. Interview transcripts were analyzed using Template Analysis. We adapted SOS and planned implementation strategies to address identified barriers.

**Results:**

Facilitators of implementation of SOS were previous quality improvement experience and engagement of clinic and administrative leadership. Barriers to implementation were a more diverse patient population, a decentralized administrative structure, and communication challenges throughout the organization. Program adaptations focused on patient instructions and educational materials as well as elimination of follow-up phone calls. Implementation strategies included early and frequent engagement with organizational leadership and a smaller pilot program before organization-wide implementation.

**Conclusions:**

Use of CFIR identified facilitators of and barriers to implementation of the evidence-based colorectal cancer screening program. Program adaptations and implementation strategies based on this study may generalize to other FQHC systems that are considering implementation of a proactive, mail-based colorectal cancer screening program.

## Introduction

Federally Qualified Health Centers (FQHCs) are primary care clinics that play a critical role in providing care to low-income and uninsured patients in the United States (1). The demand for FQHC services has increased with the implementation of the Affordable Care Act (2). FQHCs receive federal and state funding and are required to report outcomes for selected conditions annually to the Health Resources Services Administration (HRSA) as a contingency of receipt of this funding (1). HRSA requires FQHCs to demonstrate improvement in patient care in addition to measuring and reporting outcomes (1). To fulfill these requirements, FQHCs must implement efficient and effective approaches to quality improvement (3–5).

Despite the existence of effective screening tests, colorectal cancer remains a leading cause of cancer death in the United States (6). Significant disparities exist in use of colorectal cancer screening tests among low-income and minority populations, which likely contribute to disparities in colorectal cancer health outcomes in these populations (6). Although rates of colorectal cancer screening at FQHCs have increased slowly during the past 2 years, they remain well below national goals proposed by the Healthy People 2020 guidelines (1). In 2013 at FQHCs nationally, only 33% of eligible adults had evidence of current colorectal cancer screening (1); Healthy People 2020 guidelines have a target rate of 71% (7).

There is strong evidence from randomized controlled trials that mail-based fecal occult blood testing programs increase colorectal cancer screening (8,9). One such program, Systems of Support (SOS), developed and tested in an integrated health care system, demonstrated that a high rate of screening could be attained using proactive mail-based colorectal cancer screening tests (9). The original clinical setting in which SOS was developed and tested is part of a large integrated health care system in the Pacific Northwest. Approximately 80% of patients in the study were non-Hispanic white, and all were insured (9). SOS was tested in 21 primary care clinics that use a single centralized electronic health record (EHR) system and that pioneered implementation of the Patient Centered Medical Home model of care. In the original comparative effectiveness trial, 3 levels of the colorectal cancer screening program were tested. In the lowest-intensity intervention, patients received only mailed instructions and colorectal cancer screening kits to complete and return by mail. In the moderate-intensity intervention, patients who did not complete screening after the initial mailing received a reminder telephone call from a medical assistant. In the most intense intervention, patients who did not complete screening received a telephone counseling session from a registered nurse trained in motivational interviewing. All 3 levels of SOS interventions resulted in a higher proportion of patients who were current with colorectal cancer screening: 26% were screened in the usual care group, 51% were screened in the lowest-intensity SOS group, and 65% were screened in the most intensive intervention group (9). The key components of SOS are described in Table 1.

**Table 1:** Key Components of the Original Systems of Support (SOS) Colorectal Cancer Intervention and Corresponding Chronic Care Model Constructs, United States, 2014

SOS Component	Chronic Care Model Construct
Registry of patients’ current colorectal cancer screening status based on electronic health record data	Information systems
Mailing of patient information regarding colorectal cancer screening	Self-management support
Mailing of fecal occult blood test kits with stamps and reminders	Delivery system design
Medical assistant intent clarification and action planning for uncompleted testing; proactive nurse care, decision counseling, and motivational interviewing strategies for uncompleted testing	Delivery system design and self-management support
Academic detailing of colorectal cancer screening guidelines for physicians	Evidence-based guidelines and decision support
Early and ongoing identification of potential clinic policy changes to support intervention implementation and maintenance	Resources and policies

Briefly, SOS is based on the Chronic Care Model and uses EHRs to create a registry of patients who are overdue for colorectal cancer screening; these patients are provided via mail educational materials and a kit to complete colorectal cancer screening at home. For patients who do not complete the kits, SOS offers follow-up from medical assistants and registered nurses who are trained in motivational interviewing. The patient registry is created and managed centrally; all materials are mailed and processed by a single staff member. This centralization allows primary care systems to implement the intervention across multiple clinics with minimal disruption to clinic workflow.

Implementation of a program like SOS would allow FQHCs to address colorectal cancer screening without increasing the workload on individual providers or medical teams at the point of care. However, significant differences between the integrated health system in which SOS was developed and tested and FQHC settings may create barriers to implementation and potentially decrease the impact of the program. Nationally, 42% of FQHC patients are non-Hispanic white, and 28% are uninsured (10). Although most FQHCs have adopted EHR systems, use of these systems to promote quality improvement has been limited (11).

The Consolidated Framework for Implementation Research (CFIR) identifies the types of factors that influence implementation of an intervention (12). The 5 key domains included in the CFIR model are intervention characteristics, outer setting, inner setting, characteristics of individuals, and processes (12). CFIR can guide adaptation of a program like SOS in a new setting and highlight facilitators of and address potential barriers to its successful implementation (12). The CFIR model has been used widely to guide adaptation and evaluate implementation of evidence-based treatment programs in substance use disorders (13).

In this article, we aim to identify facilitators of and barriers to implementation of a proactive, mail-based colorectal cancer screening program in an FQHC setting that draws on the published evidence from the evaluation of the SOS program. We report the facilitators of and barriers to implementation of SOS in an FQHC setting based on preimplementation interviews at the site, using the CFIR model to design the interview guide, and describe the adapted colorectal cancer screening program and an implementation plan for the adapted program that addresses identified barriers. This article’s findings will inform a larger-scale dissemination and implementation plan for the adapted colorectal cancer screening program in FQHCs nationally.

## Methods

We worked with the WWAMI (Washington, Wyoming, Alaska, Montana, and Idaho) region Practice and Research Network (WPRN) to identify an FQHC organization in which we could test adaptation and implementation of the evidence-based SOS colorectal cancer screening program. The FQHC organization was selected because of its interest in colorectal cancer screening and willingness to participate. The FQHC organization comprises 7 primary care clinics, serving more than 15,000 patients annually. Approximately 90% of the FQHC organization’s patients are low-income, 30% are members of racial or ethnic minority groups, and 20% are uninsured. In 2014, only 37% of the FQHC organization’s eligible patients had completed recommended colorectal cancer screening, which is similar to the rate of colorectal cancer screening among FQHC patients nationally (10).

The principal investigator interviewed selected staff representing varied roles in the FQHC organization. Interviews were organized by job role. In a case in which only one person represented a specific job title (eg, chief medical officer), an individual interview was completed. When multiple individuals represented a job title (eg, physician), a group interview was completed. Organizational leaders (chief medical officer, chief quality officer, director of nursing, director of information technology) were responsible for overseeing operations at all 7 primary care clinics. Clinical staff interviewed (1 registered nurse, 3 medical assistants) and primary care providers (5 family physicians) worked primarily at 3 of the 7 FQHC organization’s primary care clinics but were familiar with operations and clinical workflow at all 7 sites. We used purposeful sampling of the FQHC organization’s staff to ensure that subjects could provide diverse perspectives on the FQHC organization’s structure, organizational culture, quality improvement activities, and clinical care. Subjects completed in-person interviews individually or in groups during a 1-day site visit. Two interview subjects were men, and 11 were women. The University of Washington’s institutional review board reviewed and approved all study procedures.

We developed a semi-structured interview guide based on the CFIR model (12). We created a pictograph that was used during each interview to orient participants to the general concept of SOS as it was originally designed. Specific questions to address the key domains of the CFIR model were created or identified from published literature (14). For example, “What external pressures or incentives to improve colorectal cancer screening does your organization experience?” addressed the external context, and “What are the advantages of using ‘Systems of Support’ as compared with the current approach to colorectal cancer screening?” addressed aspects of SOS (12). For all questions, follow-up probes were tailored to specific job roles and the individual’s responses to initial questions. Interviews lasted 30 to 45 minutes. All interviews were audio-recorded and transcribed by a professional transcriptionist. Participants received a $50 incentive for completing interviews.

Analysis was based on the Template Analysis model (15). We created an initial coding template that included codes for constructs of the CFIR model that the research team, which included the principal investigator (A.C.) and co-investigators (L.B. and A.E.), anticipated would be identified in the data. For example, for “external context,” a code was created for federal reporting requirements, and for “elements of the intervention,” a code was created for health information technology capacity. The principal investigator used the initial template as a guide when reviewing all transcripts. Additional codes were added after reviewing transcripts. After the initial coding, all transcripts were reviewed by a second investigator to confirm that code assignment to representative quotations was appropriate. The principal investigator and second investigator (L.B.) met to review assigned codes, identify potential discrepancies in coding, and achieve consensus in code assignment. The principal investigator organized consensus codes around key themes based on the 5 key domains of CFIR (intervention characteristics, outer setting, inner setting, characteristics of individuals, and processes).

The research team (A.C., A.E., L.B.) used the themes to develop potential modifications to SOS that would preserve the critical components of the intervention and ensure that the intervention fit well with the existing clinical context at the FQHC organization. If no modification to the intervention could be made, the research team instead created implementation processes that addressed identified barriers. We designed the final intervention, Proactive Colorectal Cancer Screening (ProCRCScreen) program, and the implementation plan from the results of this process.

## Results

The facilitators of and barriers to implementation of the original SOS intervention are outlined in Table 2. The FQHC organization’s significant previous quality improvement experience, including participation in local and national Patient Centered Medical Home initiatives (outer setting), was identified as a facilitator to implementation. The FQHC organization had also previously developed a process for pilot testing and evaluating new programs and subsequently planning for broader use of the program in the organization.

**Table 2:** Facilitators of and Barriers to Implementation of an Evidence-Based Colorectal Cancer Screening Program, United States, 2014

CFIR Construct	Facilitators	Barriers	Adaptations and/or Implementation Strategies
**Outer Setting**			
Patient needs and resources: extent to which patient needs are accurately known and prioritized by organization	Established “health access” program, which provides no-cost or low-cost care to uninsured patients	• No organized program for providing specialty and/or hospital care to uninsured patients outside of the organization • Many non–English-speaking and low literacy patients	• Work with hospital administrators and community organizations to create partnerships that could provide care for uninsured patients diagnosed with colorectal cancer through ProCRCScreen intervention • Limit initial implementation to English- and Spanish-speaking patients • Provide graphically based instructions for conducting colorectal cancer screening test as part of intervention
Cosmopolitanism: degree to which organization is networked with other external organizations	Organization participated in regional Patient Centered Medical Home initiative		
External policy and incentives: external mandates, regulations, and incentives	Organization recently became FQHC, necessitating greater emphasis on reporting and quality improvement	Currently no financial incentives for improving colorectal cancer screening rates	• Align inclusion/exclusion criteria and outcomes with those for required reports • Ensure that new systems can support quality improvement projects in other clinical areas
**Inner Setting**			
Structural characteristics: social architecture, age, maturity, and size of an organization	Previous QI experience led to development of model in which programs could be pilot-tested at a single clinic and spread to other clinics after initial evaluation	• Organization is large and decentralized • Organization recently underwent rapid growth and change in leadership structure	Initiate pilot at 2 sites and evaluate before spread
Networks and communications: nature and quality of social networks and communication within an organization	Existing meeting structure/communication strategies can be leveraged to introduce new programs	• Communication challenges across the organization and within teams in the organization identified by almost all subjects • Family Medicine Residency Training Program and Community Health Center organization share mission of providing health care, but have separate administrative and communication structures	• Create communication strategy to engage multiple levels at the practice (ie, administration, providers, and medical staff) • Research team to attend team meetings before implementation
Culture: norms and values of organization	• Individuals within the organization are committed to improving the organization • Shared mission to care for the underserved • Creation of CQO position that reports directly to CEO reflects importance of quality improvement within the organization	New programs are adopted and implemented at the discretion of administrative leadership	• Early meeting with practice leadership to introduce ProCRCScreen • Research team provides regular communication with leadership and solicits input from leadership when needed
**Implementation climate (specific to this colorectal cancer screening program)**			
1. Tension for change: degree to which stakeholders perceive current situation as needing change	Leadership has strong motivation to improve colorectal cancer screening	Clinical staff have conflicting opinions on best way to approach improving colorectal cancer screening	Research team to provide educational training (didactic presentation) to all practice staff, emphasize effectiveness of different colorectal cancer screening strategies
2. Compatibility: degree of fit between intervention and current workflow and systems	• Pieces of the intervention could fit within current workflow • New roles and workflows are consistent with leadership vision for organization	Intervention may require creation of new role (care manager)	Work closely with CQO to ensure that workflow and staffing will support implementation
3. Relative priority: shared perception of importance of implementation	Leadership voiced strong support for colorectal cancer screening as a priority and approach as a good fit for “where the organization is going”	Multiple people report “change fatigue”	Plan adapted SOS implementation to avoid overlapping with other quality improvement or practice change initiatives
4. Organizational incentives and rewards: extrinsic incentives or internal incentives for implementation	New CQO has system for providing performance reports to providers and clinical teams, which could create incentives	No financial incentives for providers or clinical teams are tied to performance	Provide colorectal cancer screening reports to participating providers before and after implementation
5. Goals and feedback: degree to which goals are clearly communicated and feedback about achieving these goals is provided	Performance reports can be created	No systematic way for sharing performance reports	• Work with CQO and providers to determine best way to share performance reports • Research team to present summary of results of ProCRCScreen program implementation in person and electronically
6. Learning climate: climate in which individuals feel safe to try new methods, sufficient time for evaluation	Multiple interviewees mentioned teaching environment as supportive for implementing new things and learning new skills	• Fast-paced clinic environment and financial pressures mean that most organizational resources are devoted directly to clinical care • Not much time or structure for clinical staff to participate in development and implementation of new programs	• Early engagement with residents and residency faculty physicians • Keep implementation as simple as possible to limit amount of training needed
**Readiness for implementation**			
1. Leadership engagement: commitment of leaders and managers to implementation	Leadership all participated in preimplementation interviews — all very enthusiastic about program and willing to be involved	• CEO not involved • Leadership and organization are not financially accountable for success of intervention	Research team to work with physician champion to engage CEO
2. Available resources: level of resources dedicated for implementation	• Existing HIT systems can be used • Grant funding to support clinic engagement and parts of implementation	Limited organizational resources may affect scalability and sustainability of the program at the practice	Implementation to be based on workflows that are scalable throughout the organization with current staffing and HIT resources
3. Access to knowledge and information: knowledge about intervention and implementation	Multiple clinical staff participated in preimplementation interviews to understand components of program and prepare for implementation	NA	Ensure frequent communication with staff before and during implementation and availability of research team to answer questions
**Individuals**			
Knowledge and beliefs about the intervention: attitudes toward and value placed on the intervention	Leadership and clinical staff voiced understanding of how the intervention works and understanding of principles on which it is based	Some clinical staff (physicians and medical assistants) had incomplete knowledge about patient preferences for colorectal cancer screening and effectiveness of different colorectal cancer screening tests	Plan academic detailing for clinical staff to provide information about evidence-based colorectal cancer screening tests
**Process**			
Planning: degree to which implementation is planned in advance	Detailed preimplementation evaluation and implementation planning done by research team in collaboration with organization	Geographic distance between research team and organization makes frequent in-person meetings difficult	• Implementation will be introduced at in-person site visit • Worked closely with CQO to develop workflow and implementation plan
Engaging opinion leaders: individuals from the organization with responsibility for implementation	• All leadership (except CEO) participated in preimplementation evaluation • Providers leading colorectal cancer screening, clinical care, and education at the organization are engaged as practice champions	Practice champions have multiple full-time responsibilities (ie, teaching and clinical care)	• Frequent direct communication with practice champion • Research team to provide tips and strategies in a format that is easy for the practice champion to disseminate through the clinic
**Intervention Characteristics**			
Intervention source: perception about whether intervention is externally or internally developed	Experience implementing program (Patient Centered Medical Home) that was developed in same integrated care system	Integrated care system in which intervention was developed viewed as significantly different from new setting	Emphasize similarities between settings and adaptability of program when planning implementation
Adaptability: degree to which an intervention can be adapted to meet local needs	NA	Original trial of SOS intervention tested 3 levels of intensity, allowing research team to evaluate individual components for cost versus benefit from the perspective of the new setting	NA
Trialability: ability to test intervention on a small scale in organization	• FQHC has previous experience testing new programs on a small scale before widespread implementation • Program easily implemented on small scale first	NA	Plan initial implementation on a small scale
Relative advantage: perception of advantage of program compared with alternatives	Benefit of new program clearly identified by most people	NA	NA

Abbreviations: CEO, chief executive officer; CFIR, Consolidated Framework for Implementation Research; CQO, chief quality officer; FQHC, Federally Qualified Health Center; HIT, health information technology; NA, not applicable; QI, quality improvement.

We used the identified barriers to plan adaptations to the original SOS program, creating ProCRCScreen. A barrier to implementation identified through interviews was limited personnel resources to conduct follow-up telephone calls (inner setting). The research team reviewed the published effectiveness data from the original SOS trial, which demonstrated that the main impact of the SOS intervention came from the mailed colorectal cancer screening kits, with more modest additional benefit from telephone follow-up (9). Therefore, the ProCRCScreen intervention does not include the telephone follow-up component for patients who have not yet completed colorectal cancer screening.

FQHC organization reporting requirements were both a facilitator and a barrier. These reporting requirements for colorectal cancer screening are elements of the outer setting that create additional incentive to implement ProCRCScreen. However, if the inclusion or exclusion criteria for the program are different from the reporting requirements, a potential barrier to implementation could be created (intervention characteristics). To leverage this facilitator and address this potential barrier, we ensured that the inclusion and exclusion criteria for ProCRCScreen matched the inclusion and exclusion criteria that the FQHC organization uses for required reporting. The required FQHC reports for measurement of colorectal cancer screening include all patients aged 50 to 75 years with an office visit in the previous 1 year, whereas the original SOS intervention included patients with an office visit in the previous 2 years (16). This change may increase the effect of the intervention if patients with less frequent primary care visits are less likely to complete recommended colorectal cancer screening.

Another potential barrier identified by the FQHC was a diverse patient population with low health literacy, limited English proficiency, and primary language other than English (internal context). To address this potential barrier, ProCRCScreen includes a graphically based brochure to help patients with limited English proficiency and primary language other than English understand the directions for completing screening.

We identified communication challenges in the organization and within teams in the organization (inner setting) as potential barriers to implementing SOS. To address these barriers, we created an implementation strategy that includes research staff attending team meetings before implementation and dissemination of both detailed and brief study materials to FQHC organization leadership and staff. Another potential barrier to implementation was the geographic distance between the research team and the FQHC organization, which makes frequent in-person meetings difficult (process). To address this barrier, we worked closely with the chief quality officer during our preimplementation site visits to develop a detailed workflow and the implementation plan.

## Discussion

In this article, we describe a systematic process, informed by CFIR, to adapt and plan implementation of an evidence-based colorectal cancer screening program. The CFIR model identified factors that could influence success of implementation when translating an evidence-based intervention to a new setting.

Our formative evaluation identified several unanticipated barriers that we addressed through a combination of program adaptations and implementation strategies. Proposed adaptations related to differences in patient population, such as use of the graphically based brochure to accommodate patients with limited English proficiency or low health literacy, and to the limited resources of the new clinical setting, such as eliminating telephone follow-up after initial colorectal cancer kit mailing. Because most FQHC organizations provide care to similarly underserved patient populations and have similar organizational resources (1), the proposed adaptations to the original SOS program may generalize to varied FQHC organizations.

In contrast, the tailored implementation strategies, such as creating communication tools and templates, required detailed knowledge of organizational structure and practice workflow. There is wider variation in these characteristics across FQHC organizations. Successful implementation of ProCRCScreen with FQHCs nationally may require at least basic knowledge of clinical workflow and infrastructure across FQHC organizations’ settings.

Widely used approaches to implementation of new programs, such as practice facilitation and learning collaboratives, provide primary care practices with opportunities to tailor implementation strategies based on local organizational structure and workflow (17,18). However both approaches are resource-intensive. Practice facilitation requires support of a trained staff member to engage with the practice in designing and implementing new programs (18). Learning collaboratives require a critical mass of participating practices and dedicated time for clinic staff to participate in meetings (17). Additionally, in systematic reviews, learning collaboratives were not consistently associated with improved patient outcomes (19). Although practice facilitation is associated with modest benefits in patient outcomes, the costs may prevent widespread adoption of this approach to quality improvement (20). Alternative approaches, such as brief preimplementation site visits by the research team and creation of common implementation tools, may offer a more efficient method to create tailored implementation plans based on local workflows.

Selection of the FQHC organization for this study was based on willingness to participate, which creates selection bias and may limit the generalizability of our findings. The participating FQHC organization may have greater readiness to implement a proactive mail-based colorectal cancer screening program compared with nonparticipating FQHC organizations. Additional barriers not identified in this study may be present in other FQHC settings. We were able to interview staff from only 3 of the 7 FQHC organization clinics, limiting our ability to assess facilitators of and barriers to implementation in the FQHC organization. However, we were able to conduct interviews with FQHC organization leadership, which provided insight into the overall organization and additional detail on aspects of each FQHC clinic. 

Despite these limitations, our formative evaluation, based on CFIR’s theoretical model, was a useful approach to plan the translation of an effective intervention into a diverse primary care setting. Using CFIR helped us identify critical facilitators of and barriers to implementation that may not have otherwise emerged. To improve population health, widespread implementation of effective interventions across diverse primary care settings is needed (21). Should the results of our formative evaluation strategy be validated on a larger scale, investigators and quality improvement experts should be encouraged to consider this approach when designing future dissemination and implementation studies.
